# Editorial: Biomarkers and therapeutic targets in the pathogenesis of neurodegenerative diseases: Functions, implications, and perspectives

**DOI:** 10.3389/fnmol.2023.1167747

**Published:** 2023-03-14

**Authors:** Junhui Wang

**Affiliations:** Sinai Health System, Lunenfeld-Tanenbaum Research Institute, Mount Sinai Hospital, Toronto, ON, Canada

**Keywords:** biomarkers, Adult Degenerative Scoliosis (ADS), non-coding RNAs, circular RNAs, neurodegenerative diseases (NDs), NMDA receptors, stroke, cerebral small vessel disease (CSVD)

Neurodegenerative diseases (NDs) present a range of conditions that are commonly characterized by the deterioration of motor, sensory, or cognitive systems. NDs are caused by the progressive impairment to neuronal cells and the loss of neuronal connectivity (Gaetano et al., 2022). Although the most common NDs are Alzheimer's disease (AD) and Parkinson's disease (PD), prion disease, femoral temporal dementia (FTD), amyotrophic lateral sclerosis (ALS), motor neuron diseases, and Huntington's disease (HD) also fall into this category. The mechanisms of NDs are not well-understood; however, generally, NDs are considered as proteinopathies with misfolded protein aggregates in cells (Brittany and Dennis, 2017). The current Research Topic includes a series of research and review articles discussing the role of small molecules (mRNA, non-coding RNAs, etc.) in NDs and their potential as diagnostic biomarkers and therapeutic targets in these diseases.

Adult Degenerative Scoliosis (ADS) is a debilitating spine condition with asymmetric spinal degeneration and multiaxial rotational deformity due to progressive degenerative changes (York and Kim, 2017). A research article from Shi et al. deployed whole-transcriptome sequencing to explore the difference between common disc degeneration and ADS, which may indicate the potential mechanism of ADS. In their study, a variety of RNAs, including circular RNA (circRNA), long non-coding RNAs (lncRNA), microRNA (miRNA), and mRNA expression profiles were investigated. Differentially expressed (DE) RNAs were conspicuously identified in the ADS group compared to their counterparts in the disc herniation group: 3,322 DE mRNAs, 221 DE lncRNAs, 20 DE miRNAs, and 15 DE circRNAs were identified in the ADS group. Most RNAs were relevant to the biological functions of endocytosis, apoptosis, etc. Therefore, this study provided evidence supporting the potential role of non-coding RNAs in ADS etiology.

He L. et al. summarized the recent progress in exploring the roles of non-coding RNAs in NDs (AD, PD, etc.), with a particular focus on circRNAs. With a closed circular structure, circRNAs are more stable in cells than their liner mRNA counterparts, and are very promising candidates for gene therapy (Wu et al., 2022). The review discussed the sponge function of circRNAs, emphasizing the fact that individual circRNA can sponge different miRNAs and actively interact with other mRNAs and non-coding RNAs. The study postulated a possible circRNA–miRNA–mRNA network which could be part of a complicated regulation system during the development of NDs.

Non-coding RNAs can also function as potential messengers between neurons and glial cells. They circulate in body fluids and are excellent for communication because of their ability to cross the blood-brain barrier. In another review article, Wang et al. delineated the role of exosomal non-coding RNAs, including miRNAs, lncRNAs, circRNAs, and PIWI (P-element Induced WImpy testis)-interacting RNAs (piRNAs), in Central Nervous System (CNS) diseases, especially NDs (AD, PD, etc.). Exosomes in the niches of the CNS have crucial impacts on the interplay between cells (neuron-neuron, neuron-glia, etc.) because of their ability to carry small molecules, including RNAs. This review summarized the recent research progress on exosomal non-coding RNAs in CNS disorders and discussed the potential of small molecules containing exosomes as diagnostic biomarkers and for therapeutic applications. Over the last decade, fluid biomarkers (soluble amyloid, tau and α-synuclein, etc.) have been a hot topic in the research of NDs, with the hope of finding diagnostic markers. Further understanding of exosomal non-coding RNAs will certainly make these molecules potential candidates in this field.

Autophagy is the major intracellular mechanism for degrading accumulated misfolded proteins. Defects in autophagy pathways have been proven be closely associated with NDs at different stages (Guo et al., 2018). Basri et al. provided another literature review of recent research on how circRNAs participate in the regulation mechanism of NDs to systematically orchestrate autophagy cascade. The idea is derived from the facts that circRNAs tendes to accumulate in the aging CNS and aging is the major predisposing factor of NDs. The interplay between circRNAs, autophagy, and the different varieties of NDs (AD, PD, ALS, HD, etc.) was profiled in this review according to recent publications.

In another report on proteome and transcriptome studies of ischemia, He J. et al. used the hippocampal neuronal HT22 cell line to study NMDA receptor (NMDAR)-mediated excitotoxicity and NMDAR hypofunction *via* an ischemic insult model or *via* deleting the receptor. The NMDA receptor plays a complex role in cerebral ischemia by exerting both pro-death and pro-survival signaling pathways (Li et al., 2022). The data presented here aimed to depict the proteome and transcriptome profiles of “hyper” and “hypo” NMDA receptors. Interesting expression patterns of protein and RNA were identified in these two groups, which provide insights for future study of the dynamic change of the NMDA receptor during ischemic insult.

Transplantation of neuronal stem cells in NDs has shown promising results and stem cell-based therapy for NDs is a current hot topic of research (Singh et al., 2016). A research article from Gupta et al. found that imidazole-based GSK-3β inhibitors could facilate the transdifferentiation of human mesenchymal stem cells (MSCs) to neurons, which provided a novel platform with a potential single-molecule formula, instead of a “chemical cocktail” solution, to induce transdifferentiation.

Cerebral small vessel disease (CSVD) is considered the most common etiology of vascular dementia or cognitive dysfunction, and it contributes to the pathogenesis of AD (Kim et al., 2020). Zou et al. performed gene differential analysis of patients with AD and CSVD from public databases to explore the underlying molecular mechanisms associated with both diseases. Differentially expressed genes (DEGs) were identified, and most DEGs were linked to endocytosis and oxytocin signaling pathways. Among the DEGs, SIRT1, an obesity- and metabolic-related gene was postulated to be a key gene. These results were reasonable since aberrant lipid metabolism is a strong risk for AD.

This Research Topic highlights the novel mechanisms of NDs by focusing on multiple types of RNAs, especially non-coding RNA and circular RNAs. The articles collected here will add significant value to further understanding of NDs *via* novel perspectives.

## Author contributions

JW designed and wrote the manuscript.
